# Supplementary material for: Are carbon emissions trading and green financial instruments synergistic? -Comprehensive quantitative research based on content analysis
Source: PLoS One. 2024 Mar 7;19(3):e0298601. doi: 10.1371/journal.pone.0298601 (PMC10919601; doi:10.1371/journal.pone.0298601)
Supplement: S1 Data — (ZIP) [file pone.0298601.s001.zip › Data/地级市Z_F工作报告.zip!/╘╞─╧/╙±╧¬╩╨/└┤╫╘╣1⁄2╓┌║┼í╛┬φ┐╦╩2╛▌═°í┐_2017.docx]

各位代表：

　　我代表市人民政府，向大会报告政府工作，请予审议，请市政协委员和列席人员提出意见。

　　一、2016年工作回顾

　　刚刚过去的一年，是玉溪发展极不平凡的一年。面对经济下行压力持续加大、卷烟产销下滑等严峻形势，市人民政府在市委的坚强领导下，在市人大和市政协的监督支持下，团结和依靠全市各族人民，认真贯彻省委、省政府重大决策部署和市第五次党代会精神，统筹推进稳增长、调结构、促改革、惠民生、防风险各项工作，综合施策、精准发力，经济保持平稳发展，社会保持和谐稳定，较好完成了市四届人大四次会议确定的主要目标任务，实现了“十三五”良好开局。预计全市完成生产总值1309亿元、增长8%左右，一般公共预算收入131.1亿元、增长5%，规模以上固定资产投资868亿元、增长30%，社会消费品零售总额326.3亿元、增长12%，城镇居民人均可支配收入32150元、增长8.5%，农村居民人均可支配收入12040元、增长9.7%，城镇化率48.5%，居民消费价格总水平上涨1.2%，城镇登记失业率3.5%，单位生产总值能耗下降3.2%。

　　一年来，重点抓了7个方面的工作。

　　——我们把稳增长作为第一要务，经济发展稳中向好。深入开展“争先创优跨越发展”大讨论大行动和作风转变年、工作落实年、创新发展年活动，集合政策，整合资金，加大对重点产业、重点项目支持力度，经济实现稳定增长。强化政策支持。认真落实省政府稳增长政策，制定39条具体措施，整合安排6.9亿元用于产业发展和项目建设，统筹调度煤电油运，支持企业扩销促产、电力市场化交易、降本增效，节约电费9.3亿元、运费4050万元，减免企业税收39.5亿元。建立融资保证金池、企业调头资金池，引导银行发放园区基础设施建设贷款2.3亿元，帮助企业调头到期贷款6.4亿元。强化产业发展。实行烟草下滑非烟补、二产不足一产三产补、工业不足建筑业补、规上不足规下补，三次产业比重由10.2∶55.0∶34.8调整为10.2∶51.3∶38.5，第一产业和第三产业增速全省第一。稳步发展农业，实现增加值135.7亿元、增长6%。粮食十一连增，烤烟减量不减收，蔬菜、林果、花卉等特色产业提质增效，畜牧业产值增长3%。聚力抓好工业，规上工业增加值590亿元、增长3.7%。全力支持红塔集团发展，卷烟及配套产业实现增加值390亿元、下降5%左右；大力发展非烟工业，增加值达200亿元、增长12.5%，矿冶及装备制造、生物医药及大健康产业增加值分别增长9.2%、7.2%。推进生物医药、重庆东恩等产业园建设，年产30亿个液体包装盒、3万吨数控机床铸件等35个工业项目建成投产。新增2000户小微企业，新纳规工业企业86户、总数达412户，6户企业在中小板、新三板挂牌上市，民营经济增加值增长8%。推动房地产业健康发展，建筑业增加值53.6亿元、增长25%。成功引进万达集团，开启商业运营新模式。大力发展批发零售、住宿餐饮等服务业，第三产业实现增加值502.9亿元、增长13%。发展壮大文化旅游产业，仙湖时光栈道完工，戛洒哀牢小镇提档升级，寒武纪乐园、仙湖山水等项目顺利推进，广龙旅游小镇开工，旅游总收入增长16%。促进信息消费，发展增值服务，华为云计算数据中心、华唐大数据服务外包基地正式运营，云南中医药大数据中心上线，信息服务业用电量超过1亿千瓦时、增长12%，信息产业增加值增长7.5%。推动物流业提档升级，通力物流、九溪润特等项目快速推进，现代物流业增加值增长4.7%。强化科技支撑。新增院士专家工作站3个、市级技术研究中心5个、高新技术企业21户、科技型中小企业30户，科技贡献率达58.5%。强化投资拉动。落实重点项目副市长组长负责制，压实项目推进责任，46个省级、340个市级“四个一百”重大项目分别完成投资82.7亿元、320亿元。推进52个省级“三个一百”工业转型升级项目建设，完成投资160亿元。强化资金保障。争取上级财政转移支付资金109亿元、省级债券转贷资金189亿元、国家专项建设基金6.3亿元、省级重大项目建设投资基金42.7亿元，40个PPP项目列入财政部储备库。设立政府投资母基金。引导金融机构支持地方发展，新增贷款57.3亿元。

　　——我们把五网建设作为头等大事，基础瓶颈实现突破。制定实施五网建设五年行动计划，投入3.8亿元前期工作经费，强化现场推进，加快167个重大项目建设，完成投资188亿元。路网建设全面提速。昆玉电气化铁路竣工通车，玉溪迎来高铁时代。玉磨铁路征地拆迁基本完成，工程建设加快推进。武易高速玉溪段、昆明绕城高速东南段主体工程完工，晋红、江通高速进展顺利，澄川、大戛、元蔓和弥玉、玉楚高速试验段开工建设，国道213线、省道313线改造加快推进，改扩建农村公路1671公里。水网建设扎实推进。大龙潭引水工程向星云湖补水1000万方，苗茂、鲁布2座中型水库和8座小型水库建设进展顺利，病险水库除险加固58座，解决了15.3万农村人口饮水安全问题。新增高效节水灌溉面积15.5万亩。能源网建设力度加大。中石油玉溪油库主体工程完工，建成天然气管道支线171公里、城市燃气管道110公里，推广使用天然气585万立方米。库容18亿立方米、装机27万千瓦的戛洒江一级水电站开工，建成4座风电场、3座光伏电站，10座输变电站建成投产，电动汽车集中式充电站开工。互联网建设全面推进。加快信息惠民、宽带中国、宽带乡村、智慧城市试点建设，新增全光网端口27万个，新建移动基站1100座，行政村实现光纤宽带和4G全覆盖。“互联网+”广泛应用，区域卫生信息平台基本建成，621所中小学教育云服务终端投入使用。航空网建设正式启动。先后与美中基金、广东龙浩、昆钢集团、雪域飞鹰运动航空签订产业战略合作协议，江川、新平、元江通用机场前期工作启动，南方航空护林总站直升机场完成主体工程。

　　——我们把改革开放作为强大动力，发展活力日益增强。认真落实市委改革部署，推进重大改革措施落地，重点领域和关键环节改革取得突破，改革红利逐步释放。深化“放管服”和商事制度改革，推行“双随机、一公开”监管工作，“五证合一、一照一码”新登记注册企业1.6万户。全面实施供给侧结构性改革，化解和淘汰钢铁产能291万吨、平板玻璃60万重量箱，消化库存商品房120.5万平方米。扎实推进农村重点领域改革，完成139万亩土地承包经营权确权登记颁证，新增土地流转3万亩。市商业银行完成增资扩股、更名为云南红塔银行，通海、易门农信社改制为农商行。“营改增”、不动产统一登记全面推开。实施抚仙湖资源环境管理体制改革，径流区实现统一托管。公立医院改革深入推进，城乡医保整合、分级诊疗、DRGs付费制度等改革走在全省前列。机关公务用车制度改革完成，工资分配、养老保险、人才培养、科技体制、殡葬制度、社会治理等改革成效明显。成功举办“相约春天”“收获金秋”等大型招商活动，引进市外国内资金800.4亿元、增长20%，利用外资117万美元。加强国际经贸合作，设立玉溪驻泰国、老挝、越南、柬埔寨商务代表处，新增进出口实绩企业49户、境外直接投资项目12个，实现进出口总额21.4亿美元、增长13%。

　　——我们把城乡建设作为重大任务，城乡面貌明显改观。启动玉溪城市总规修改，完成了中心城区建筑风貌导则、地下空间开发利用规划、城市地下综合管廊专项规划编制和城乡规划管理技术规定修订，“多规合一”试点顺利推进。“六城同创”取得成效，国家节水型城市通过专家现场考核验收，列为国家海绵城市、省级地下综合管廊试点，实施海绵城市4个项目包建设，开工建设市政道路31.2公里、地下综合管廊19公里、安全骑行车道38公里，启动玉溪城市规划馆建设。玉水金岸、新天地、临岸三千城等项目建设有序推进，城市生活垃圾焚烧发电、排水管网二期加快推进，夜景照明、增绿添色景观改造成效初显。完成江川撤县设区。开展了澄江、峨山县城扩容提质试点。全面启动“点亮玉溪”和新一轮提升城乡人居环境行动，中心城区4条黑臭水体治理开工，15个建制镇“一水两污”工程完工4个，启动12个美丽乡镇建设，拆除临违建筑8万平方米，新建改造城市公厕74座。“百村示范、千村整治”开工665个、完工440个，城镇棚户区改造房屋征收58.4万平方米，农村危房改造开工40613户、竣工21757户。

　　——我们把生态建设作为立市之基，环境保护持续加强。落实环境保护“党政同责”和“一岗双责”，制定实施水污染、大气污染防治工作方案，生态环保工作得到加强。全力配合中央环境保护督察工作，全面落实整改要求。《云南省抚仙湖保护条例》修正实施，完成“三湖”保护治理“十三五”规划编制。抚仙湖北岸生态湿地建设进展顺利，居民搬迁安置房、生态展示中心建成。村落污水收集、生态调蓄带、主要入湖河道治理等一批工程治理项目投入使用，各项非工程措施落实到位，抚仙湖在全国水质良好湖泊绩效评价中排名第一。加强星云湖、杞麓湖保护治理，启动实施环湖截污治污、底泥疏挖处置、村落环境整治、农田减肥增效等项目，水质逐步得到改善。绿汁江、戛洒江、南盘江综合治理和集中式饮用水源地污染防治深入推进。启动林业生态红线划定工作，完成营造林26.6万亩、退耕还林6.2万亩。土地开发整理新增耕地393公顷，治理水土流失面积287平方公里。新建机动车尾气检测线11条，淘汰黄标车、老旧车12752辆，实施重点减排项目102个，节能减排任务圆满完成。华宁、峨山、新平创建省级生态文明县、22个乡镇创建国家级生态乡镇、13个乡镇创建省级生态文明乡镇通过考核验收。

——我们把改善民生作为最终目的，人民生活显著改善。加大民生投入，补齐民生短板，10件惠民实事全面完成。抓好整乡整村推进、易地扶贫搬迁等项目实施，基本实现6个贫困乡镇摘帽、58个贫困行政村出列、4万农村人口脱贫。完成4个移民新村建设。以创业促就业，新增城镇就业2.55万人，帮助7692名困难人员实现就业，农村劳动力转移就业43.9万人次。加强城乡社会保障体系建设，提高新农合和城乡低保补助标准，改扩建乡镇敬老院5个，建成居家养老服务中心20个、农村幸福院70个。统筹推进学前、义务、高中、高等各阶段教育发展，新建美丽校园65所，校安工程圆满收官，“全面改薄”工程加快推进，数字化校园实现全覆盖，玉溪卫校迁建工程开工，5个县区被认定为国家、省义务教育发展基本均衡县。健全完善城乡医疗卫生体系，市医院改扩建、市急救中心主体工程完工，完成2个乡镇卫生院、10个村卫生室建设，市儿童医院开工，健康环境持续改善，被列为全国健康城市试点。文化广电事业蓬勃发展，完成“三台”改革合并，建成4个乡镇文化站和6座广播电视高山发射台，文化广播影视传媒中心开工，澄江化石博物馆主体完工，江川甘棠箐旧石器遗址入选2015年全国十大考古新发现，荣获“中国楹联文化城市”称号。国家体育产业联系点工作有序推进，成功举办省第三届宗教界体育运动会暨文艺汇演等国内外赛事。村（居）委会换届选举顺利完成。加快民族地区经济社会发展，依法管理宗教事务，民族团结、宗教和顺局面进一步巩固。加强食品药品安全监管，深入开展隐患大排查、打非治违、重点行业领域专项整治，安全生产形势总体稳定。加强社会治安立体化防控体系建设，积极化解信访积案和各类社会矛盾纠纷，严厉打击电信网络诈骗、非法集资等违法犯罪行为，提升了市民安全感。深入开展“双拥”创建活动，支持国防和军队改革，全国双拥模范城实现“四连冠”。妇女儿童、残疾人、老龄、红十字、关心下一代等事业健康发展，统计、外事侨务、应急管理、防灾减灾、人防、保密、档案等工作成效明显。

——我们把依法行政作为重要保障，自身建设得到加强。认真学习贯彻全面推进依法治国若干重大问题的决定，扎实开展“两学一做”学习教育，依法从严治政，干部队伍作风明显好转。自觉接受市人大法律监督、工作监督和市政协民主监督，人大代表建议和政协提案解决率分别达50%和51.06%。启动“七五”普法，严格执行重大行政决策程序和责任追究办法，集体学法2次、重大决策听证99次。启动首个地方性法规立法工作，《玉溪市新平哀牢山县级自然保护区条例（草案）》通过市政协立法协商、市人大常委会审议。强化监察审计，驰而不息反对“四风”，查处违反中央八项规定精神问题12起，“三公”经费支出下降13.6%。强化政府党组党风廉政建设主体责任，落实领导干部“一岗双责”，开展廉洁从政警示教育。强化重点工作跟踪问效，行政问责194人，政风建设取得实效。

　　各位代表，回顾去年工作，经济社会发展成效明显，成绩来之不易，这是市委坚强领导，市人大、市政协监督支持，全市人民共同努力的结果。在此，我代表市人民政府，向全市广大干部群众，向人大代表和政协委员，向各民主党派、工商联、人民团体和社会各界人士，向中央和省驻玉单位、驻玉军警部队官兵，向各位离退休老领导，表示崇高的敬意和衷心的感谢！

　　在肯定成绩的同时，我们也清醒地看到，当前经济社会发展还存在很多困难和问题：一是产业结构不合理、工业投资不足、新兴产业培育滞后，加之卷烟产销形势仍然严峻、供需矛盾突出，稳增长压力加大。二是由于市场需求疲软、原材料价格上涨，企业生产成本上升、库存增加、利润降低，生产经营困难；部分金融机构抽贷压贷停贷现象较为突出，民营企业融资难融资贵。三是财政收支矛盾十分突出，税源不足，保工资、保运转、保民生等刚性支出加大；债务管理、还本付息压力较大。四是项目前期工作滞后，重大项目储备不足、成熟度不够，土地、资金等瓶颈制约依然突出，企业投资信心不足，投资增长乏力。五是“三湖”生态保护形势严峻，治污减排压力较大，城乡环境治理任务艰巨。六是民生领域补齐短板的任务繁重，城乡居民就业渠道不宽，持续增收困难，脱贫攻坚任务艰巨。七是有的领导干部适应经济发展新常态的能力素质不强，精气神不足，勇于担当不够，精力不集中，重点不突出，破解发展难题办法不多。我们将坚持问题导向，做好应对更加困难复杂局面的思想准备和工作准备，切实解决好发展中的各种问题，干在实处，走在全省前列，不辜负全市人民的重托。

　　二、2017年工作总体要求

　　2017年是实施“十三五”规划的重要一年，是供给侧结构性改革的深化之年，也是本届政府任期的最后一年。做好今年的政府工作，一定要认真学习领会中央经济工作会议精神，准确把握国内外经济形势和自身发展环境条件。当前，世界经济仍在深度调整，我国经济发展新常态特征更加明显，我市正处在新旧增长动能转换的关键时期，深化改革任务艰巨，加快发展不确定因素增多。但也要看到有利条件：随着国家“一带一路”、长江经济带、孟中印缅经济走廊等重大战略的推进，为加快发展提供了强大推动力；我省建设民族团结进步示范区、生态文明建设排头兵、面向南亚东南亚辐射中心，推进滇中城市经济圈一体化发展，玉溪的地位和作用更加凸显，发展的潜力和空间倍增；市第五次党代会描绘了全面建成小康社会宏伟蓝图，产业发展更加聚焦，重大项目加快推进，基础条件明显改善，内生动力持续增强。我们有信心、有能力、有条件保持经济社会持续健康发展。

　　政府工作的指导思想是：深入学习贯彻以习近平同志为核心的党中央治国理政新理念新思想新战略，全面落实中央经济工作会、省第十次党代会、市第五次党代会、市委五届二次全会和市委经济工作会精神，坚持稳中求进工作总基调，积极适应和引领经济发展新常态，以提高发展质量和效益为中心，以供给侧结构性改革为主线，主动融入国家和省的发展战略，坚持经济社会发展“5577”总体思路，聚焦关键抓改革强引领，全力以赴抓产业促跨越，精准发力抓项目扩投资，打好“三大战役”兴实体增动力，规划引领建城乡添魅力，坚定不移促转型保生态，推进经济平稳健康发展和社会和谐稳定，加快建设区域性中心城市、科教创新城市和生态宜居文明幸福的魅力之城，奋力闯出一条具有玉溪特色的跨越式发展之路，为在全省率先全面建成小康社会奠定坚实基础。

　　经济社会发展目标建议为：生产总值增长8.5%左右，规模以上固定资产投资增长25%以上，一般公共预算收入增长4%以上，社会消费品零售总额增长12%以上，城镇居民人均可支配收入增长9%，农村居民人均可支配收入增长10%，居民消费价格涨幅控制在3%以内，城镇登记失业率控制在4%以内，城镇化率达50%，万元生产总值能耗完成省下达目标。

　　做好今年的政府工作，必须牢牢把握5个着力点：一是必须坚持稳中求进的工作总基调。要保持发展定力，盯住经济发展目标不动摇，把“稳”的重心放在烟草产业上，稳住卷烟产销规模、稳住烟叶种植规模，争取贡献不下降；放在振兴实体经济和发展质量上，稳住扶持政策、稳住资金供应，确保存量企业正常生产经营。把“进”的重点放在扩大投资规模上，加快续建项目竣工，新上一批重点基础设施项目，确保投资快速增长；放在重点工业行业的转型升级上，扶持一批成长性好的新兴产业，尽快形成新的产能，带动经济增长。二是必须坚持供给侧结构性改革这一主线。深入推进“三去一降一补”，努力解决实体经济产品供需失衡、金融信贷避实就虚、房地产和实体经济失衡问题。落实“去”的任务，妥善处理去产能与强产业的关系，淘汰落后产能，加快产业转型升级；妥善处理去库存与发展房地产业的关系，坚持“房子是用来住的、不是用来炒的”定位，合理运用土地、金融、城镇化等政策工具，千方百计减少库存商品房和保障房，促进房地产市场健康发展；妥善处理去杠杆与筹资金的关系，科学合理举债，扩大直接融资，满足建设资金需求。精准“降”的举措，在降低电价、交易、税负、用地等方面成本上发力，让利实体经济，共度发展难关。做好“补”的文章，因地制宜、实事求是，逐步补齐基础设施、产业发展、脱贫攻坚、生态环境、城乡建设、社会建设6大短板。着力壮大实体经济，大力发展现代服务业，供需两侧同时发力，着力解决好培育促进消费、扩大有效产品供给等问题，提高全要素生产率和潜在增长率。三是必须坚持“三区一港”产业发展定位和“四带多园”总体布局。坚持规划引领、基金引导、市场主体推动，强化投资拉动、创新驱动、金融促动，加快推进“三区一港”建设。以园区为载体、交通为基础、产业为支撑，强化招商引资，突出抓好“四带多园”规划建设，昆玉—玉元经济带重点发展通道经济，“三湖”生态经济带突出发展绿色经济，滇中高速环线经济带统筹发展特色经济，红河谷—绿汁江热区产业经济带着力发展热区经济，打造经济“升级版”，带动县域经济快速发展。四是必须坚持科教引领创新发展。围绕“开放、创新、生态、宜居、宜业”定位，走高端化的科教创新发展之路，打造滇中地区科教创新城。认真开展“科教引领创新发展”大讨论大行动，加快规划编制，出台实施意见，制定行动计划，高起点、高标准、高效率推进科教创新发展。实施高技能人才振兴计划和职业技能提升行动计划，集聚创新创业人才和团队。突出企业主导地位，支持企业、高校、科研院所、创客协同创新，鼓励企业建设科技创新平台，加快高新众创空间、互联网产业创业园发展，启动“双创”中心建设，推动大众创业、万众创新。五是必须坚持统筹城乡协调发展。加快“三湖”生态城市群建设，做强主城、做精节点、做优县城、做特集镇、做美乡村。坚持四化同步，着力构建城乡一体产业体系，吸纳农村人口有序向城镇转移。推动城乡土地、资金、劳动力等要素平等交换，合理配置教育、卫生、文化等公共资源，推动基础设施建设向农村延伸、公共服务向农村覆盖。强化生态环境保护，加强水体、大气、土壤污染防治，保护绿水青山，守住蓝天白云，争当全省生态文明建设排头兵。

　　三、2017年重点工作

　　今年要重点抓好10个方面的工作。

　　（一）全力以赴促进经济增长

　　合力打好“三大战役”。打好民营经济战役，严格执行“非禁即入”投资政策，构建“亲”“清”新型政商关系，加大支持力度，激发和保护企业家创业热情，鼓励民间资本参与产业发展、基础设施、城镇化、社会民生等领域的投资，确保民营经济增加值增长8%。打好县域经济战役，发挥县区主体作用，突出县区资源禀赋，实行错位竞争、差异发展，每个县区培育2—3个主导产业，因地制宜打造工业强县、农业大县、旅游强县，确保各县区生产总值增速均达两位数以上，确保易门县经济总量突破百亿元。打好园区经济战役，加快工业园区规划修编，科学定位园区主导产业，全面实施实体化改革，抓好生物医药、重庆东恩、北京顺义等园中园建设，促进11个园区建设发展，确保园区增加值增长7%以上；完善园区基础设施，加快园区“三通一平”、定制标准化厂房等基础设施建设，确保园区收储土地1.2万亩，提供熟地8000亩。

　　全力增加有效投资。实施固定资产投资争先创优两年行动计划，抓好十大重点领域投资，确保全市投资突破千亿元大关。实行重点项目市级领导联系负责制，坚持项目、进度、问题、责任清单管理制度和交办制度，挂图作战、销号管理，扎实推进省57个、市535个“四个一百”重点项目建设。优化投资结构，加快238个工业项目实施，确保非电工业投资增长15%以上；加快145个五网重大项目建设，确保完成投资278亿元以上；实施PPP项目，激活民间投资，确保投资比重达40%。抓实前期工作，增加好项目大项目储备。鼓励进入玉溪参与重大项目建设的企业设立独立法人机构，确保新开工项目及时入库统计、总部经济税收合理划分、跨区项目税收应收尽收。

努力扩大消费需求。落实国家扩大旅游、文化、体育、健康、养老、教育培训等消费领域意见，深入开展十大扩消费行动，确保社会消费品零售总额达359亿元。认真落实促进全市房地产业平稳健康发展的实施意见，合理运用供地、信贷、税收等手段，加强房地产市场调控，鼓励住房消费，避免新增库存。搞好餐饮美食街区规划建设，打造特色餐饮街区，带动餐饮消费。畅通城乡销售网络，完成8个乡镇集贸市场建设，促进城乡消费。大力发展电子商务，推进通海、元江全国电子商务进农村综合示范及阿里巴巴“千县万村”工作，培育壮大骨干企业，促进线上线下消费融合发展。

　　发挥出口带动作用。加快出口基地建设，大力发展加工贸易，鼓励农产品加工企业到市外建立原料基地，建立海外营销中心，巩固蔬菜、水果等产品出口优势，扩大生物制药、高新机电等产品出口规模，确保进出口总额增长10%以上。

　　着力强化保障措施。树立吃饭靠财政、建设靠融资的理念，集中财力保工资、保运转、保民生，科学合理运用金融工具，着力解决建设资金难题。抓好项目包装，全力争取上级预算内资金、国家和省专项建设基金支持；用好政府投资母基金，合理设立子基金，重点解决企业调头转贷资金困难；加大“引银入玉”力度，加强银政企合作，用好信用再担保政策，新增贷款90亿元以上。充分发挥合和集团、红塔银行在投资发展中的重要作用。强化政府债务管理，积极争取省级新增置换债券额度，制定实施市级债务管理办法，严控举债成本和债务风险。调整完善土地利用总体规划，完成全域永久基本农田划定，清理处置闲置土地，确保供地率达70%。加强形势研判和分析预警，搞好煤电油运保障，提高经济运行质量。

　　（二）打好五网建设攻坚战

　　提速建设路网。加快江通、澄川、大戛、元蔓高速公路建设，推进弥玉、玉楚全线开工，力争永金高速新平至元江段开工建设，做好红江、晋易、澄华、新临高速戛洒至者东段等项目前期工作，确保晋红高速5月底前通车。加快国道213线改造，实施重要国省道改造计划。配合做好玉磨铁路建设，启动呈贡—澄江—江川—红塔城际铁路建设，抓好环抚仙湖轨道交通建设前期工作。

　　织密城乡水网。实施大龙潭引水配套设施工程，配合做好滇中引水工程前期工作。实施华宁矣则河、新平洋发城2座小（一）型水库改扩建工程，开工建设通海木格、峨山筇川等6座小（一）型水库，加快元江鲁布水库建设，完成易门苗茂中型水库和4座小（一）型水库主体工程，做好24件水源工程和6件引调水项目前期工作。

　　加快建设能源网。加快永济220千伏、哨坡110千伏输变电工程建设，完成4座35千伏以上输变电站建设和小城镇、中心村农网改造升级，完成6个输变电工程及城农网项目前期工作。建成光伏发电站3座、充电站5个。加快戛洒江、麻洋河一级水电站建设。推进红河天然气支线管道、市应急气源储备中心建设。

　　完善提升互联网。推进云南联通玉溪数据中心项目建设，改造新建1000个通信铁塔配套基站，扩容互联网出口带宽，实现城乡光纤网络全覆盖。建成市民服务一卡通软件平台、一站式信息惠民平台、大数据市级交换平台，扩大教育云平台覆盖面，推动中国大数据与呼叫外包服务中心落地。加快4G网络等新一代移动通信网络建设，扩大政府公共免费无线上网覆盖范围。

　　抓紧建设航空网。加强与云南机场建设集团、合和集团、龙浩集团合作，力争江川、澄江、元江通用机场开工建设，加快华宁、新平通用机场建设前期工作。

　　（三）打响工业转型攻坚战

　　推进重点产业发展。实施中国制造2025玉溪行动计划，着力调结构、转方式、稳存量、扩增量，引导企业依靠科技创新提质增效、通过产品创新调整结构，确保工业增加值增长6%以上。稳定发展卷烟及配套产业，全力配合红塔集团搞好车间技改等项目建设，优化品牌结构，稳定生产销售，支持创新新材料公司整合卷烟配套企业。加快矿冶及装备制造产业转型发展，引导玉钢集团、新兴钢铁等企业提档升级，支持贵研铂业、太标集团、蓝晶科技等企业成长壮大，抓好粗铜、数控机床、风电法兰盘等项目实施，促进杭萧钢构、国动网络通信铁塔、升华电梯落地，确保增加值增长10%以上。大力发展生物医药及大健康产业，支持沃森、维和等企业做大做强，抓好新型宫颈癌疫苗、治疗性单抗药物产业化等项目实施，确保增加值增长15%以上。实施质量强市战略，打造自主品牌，推进专利质押融资试点，大力发展高新技术产业，新增高新技术企业5户、科技型中小企业10户以上，建成市级以上企业技术中心8个。

　　突出抓好产业招商。把招商引资作为经济工作的生命线，落实“四个一再”要求，依托南博会、沪滇合作、友好城市等平台，瞄准长三角、珠三角、京津冀，关注高精尖、聚焦500强、紧盯省属大企业，实施产业招商、精准招商，力争每个县区引进2个以上大项目，每个产业培育2户以上大企业。策划包装150个以上重大项目，精心组织招商活动，确保实际利用市外国内资金增长11%以上、外资增长5%以上。强化项目落地考核，推动签约项目落地、存量项目竣工，确保红塔区、江川区、新平县、易门县和高新区新开工投资3000万元以上工业项目不少于5个，其他县区不少于3个。

　　加大帮扶企业力度。坚持市级领导联系重点民营企业制度，一企一策帮助企业解决难题。抓好扩产促销、电力市场化交易、清费减负等措施落实，促进企业降本增效。建立“政银担”风险分担机制，发挥融资保证金池、扶持工业园区及中小微企业调头资金池作用，引导金融机构脱虚入实、支持实体经济发展。发行小微企业集合债，新增新三板挂牌企业1户以上。实施中小企业成长、“两个10万元”微型企业、民营“行业小巨人”培育工程，强化企业纳规工作，力争2户成长型企业进入大企业行列。

　　（四）切实抓好“三农”工作

　　打赢脱贫摘帽攻坚战。把脱贫攻坚作为最大的政治任务、最大的民生工程，动员全市上下攻坚拔寨，全面落实领导责任和部门帮扶责任，压实县区主体责任，扎实开展“挂包帮”“转走访”，认真落实“六个到村到户” 和“五个一批”扶贫措施，做到“六个精准”，完成整乡整村推进和建档立卡贫困户易地扶贫搬迁1万人。大力推进产业扶贫，推动每个贫困乡、村形成特色产业和拳头产品。加快贫困村组公路建设，新建、改建公路2000公里。提升贫困人口劳动力素质，增强自我发展能力，培训1万人次、转移5000人。发放扶贫小额信贷资金4亿元。聚力“直过民族”脱贫，完成4个行政村、49个自然村整族推进任务。确保3个贫困乡摘帽、17个贫困行政村出列、33751名贫困人口脱贫，在全省率先实现全面脱贫目标。

　　做强特色优势产业。认真贯彻中央一号文件精神，深入推进农业供给侧结构性改革，以开放型农业为引领，加快高原特色现代农业发展，确保农业增加值增长6%以上。稳定粮食生产，搞活粮食流通，提高粮食安全保障能力。种植烤烟57万亩、收购烟叶153万担，巩固蔬菜种植面积120万亩，保持鲜切花面积3万亩、生物药原料6万亩，新增水果2.5万亩；支持希望集团推进100万头生猪和2000万只肉鸡养殖加工项目建设，抓好重大动物疫病防控，新建畜禽标准化示范场3个。实施名特优新农产品培育工程。推动玉溪国际农产品交易中心项目落地，精心办好中国蔬菜产业大会。

　　加快产业融合发展。全面实施农村一二三产业融合发展，破解农业设施用地瓶颈，大力培育新型农业经营主体，扶持龙头企业发展深加工、延伸产业链，积极发展农业装备制造，着力发展体验休闲观光农业，提高农业综合效益。新增农民专业合作社30个，新建市级家庭农场示范场100个，培育新型职业农民1600人，培训农村劳动力1.5万人，扶持发展农业小巨人20户，新认定省市级龙头企业5户，新建市级休闲农业与乡村旅游示范企业5户。

　　发展生态循环农业。创建国家农业可持续发展试验示范区，全面实施农业有害生物防治和测土配方施肥，严格控制化肥、农药使用，启动通海县规模化生物天然气项目建设，建成种养结合循环养殖示范场10个，推广稻田养鱼3万亩。加快农产品质量安全追溯体系信息平台建设，创建出口农产品食品国际认证标准管理示范区，新增“三品一标”农产品8个、云南名牌农产品3个。

　　改善农村发展条件。启动县乡公路改造提升工程，新建、改建农村公路2614公里，实现建制村100%通硬化路、90%以上通客运班线。改造中低产田地16.7万亩，建设高效节水减排面积20万亩、大型沼气池2座。病险小坝塘除险加固400座，解决15万农村人口饮水安全问题。抓好水库移民后期扶持发展工作。深化农村综合改革，稳步推进农村土地“三权分置”和经营权流转，加快推进农村集体建设用地使用权调查确权，做好易门县房地一体确权登记颁证试点工作，实施国有林场、水利投融资及水价改革。完成全国第三次农业普查。

　　（五）大力发展现代服务业

　　做强文化旅游产业。强化全域旅游发展理念，实施旅游产业转型升级三年行动计划，推进红塔工业旅游，建设精品旅游线路，打造“帽天山、抚仙湖、花腰傣”三张名片，强化宣传营销和市场监管，确保旅游总收入增长16%以上。发挥市级文化旅游投资平台作用，抓好澄江寒武纪乐园、抚仙湖山地公园、澄江化石地博物馆等35个重大旅游项目建设，加快特色旅游城市、旅游强县、旅游名镇、旅游名村建设，推进立昌、广龙旅游小镇建设，力争抚仙湖创建为国家级旅游度假区。实施体育产业发展规划，积极发展陶、铜、银、刺绣等文化创意产品，推动旅游与文化、体育、医疗、农林等产业融合发展。

　　加快发展信息服务业。支持华为玉溪云计算数据中心运营发展，深化与阿里巴巴、腾讯、亿赞普、永兴元、中国网安等知名企业合作，培育智能制造、智慧健康、3D打印、跨境电商及服务外包等产业，确保增加值增长20%以上。实施“云上云”行动计划，推进大数据开发应用，延伸“互联网+”领域，发展增值服务。推动滇中城市经济圈同城数据备份中心、南亚东南亚小语种云中心等项目建设，搭建智慧旅游、智慧交通、智慧城市等服务平台，打造泛亚丝路数据谷、“三湖”国际云计算产业园品牌。

着力培育现代物流业。制定实施加快现代物流业发展意见，推动“一核、两带、七节点”空间布局和建设发展，打造滇中城市经济圈南枢纽，确保增加值增长10%以上。加强与贵阳货车帮、上海新跃等物流企业合作，建设玉溪智慧物流信息平台，完善物联网。依托昆钢、龙浩等战略合作伙伴，加快研和钢铁、通海农产品、江川航空等物流园区规划建设，抓好九溪润特、活发物流、玉交物流等项目建设，完成通力物流、食品商贸仓储物流港建设。大力培育冷链物流运输企业，推进物流标准化试点示范工作。

　　大力发展高端服务业。实施服务经济“倍增”计划，确保第三产业增加值增长12%以上。改造提升餐饮、住宿、商贸等传统服务业，推动服务业模式创新、跨业融合。加快发展教育、科技、金融等城市高端现代服务业，丰富城市内涵，提升城市档次，打造现代服务业发展新高地。

　　（六）全面推进改革开放

　　深化各项改革。持续推进“放管服”改革，实行权责清单动态管理，统一规范市县行政许可目录，搞好行政许可事项“接管服”，强化事中事后监管，改革规范中介服务，提升政务平台综合服务能力。深化商事制度改革，全面推行企业登记“五证合一、一照一码”和个体工商户登记“证照合一”。持续推进“三去一降一补”，化解淘汰化工产能28万吨、水泥91万吨，消化库存商品房120万平方米。深化财税金融改革，建立财政事权和支出责任制度，完善县乡财政管理体制，搞好税收征管体制改革，完成县级信用社改制为农商行任务。完善城市管理体制，强化规划管理，推进综合执法，提高精细化、人性化、智能化水平。做好抚仙湖综合行政执法体制改革试点。搞好领导干部自然资源资产离任审计试点。开展事业单位及国有企业公务用车改革。进一步深化和完善公立医院改革，推进DRGs付费制度、“互联网+医疗健康”、药品耗材采购等重点工作，理顺公立医院管理体制，走出一条具有玉溪特色的新医改之路。深化教育、电力、价格、国企、事业单位等领域改革。采取有力措施，加快改革举措落地见效。

　　强化开放合作。发挥驻外商务代表处作用，引导鼓励企业“走出去”开展水电、农业、商贸等投资合作，促进外经外贸联动发展。围绕建设面向南亚东南亚国际内陆港目标，完成综合保税区规划修编和可研编制，推进申报国家级综合保税区工作。积极融入滇中城市经济圈建设，加快昆玉一体化进程，主动承接昆明教育、科技、会展等部分城市功能，承接信息、仓储、物流等产业转移。加强与北京顺义友好合作，加快与广东佛山、江苏南通建立友好城市，拓展合作领域，实现互利共赢。

　　（七）统筹城乡规划建设

　　加强规划管理。加快城市总体规划修改。完成美丽玉溪行动规划暨中心城区近期建设规划编制，编制海绵城市技术导则、县区海绵城市专项规划和乡村建设规划。加强城乡空间开发管制，开展江川区控规一张图编制，全面推行“多规合一”，完成红塔区、易门县试点任务。做好全国城市设计试点和创建省级城市规划示范点工作。加强城乡规划和管理技术规定执行监管，建立城乡规划督查制度和社会参与监督机制，提高规划权威性和严肃性。

　　加快城镇建设。做强中心城区，调整玉枕山片区规划，启动科教创新城建设，加快高铁新城、城市规划馆、城市路网美化绿化亮化等“十大工程”建设。启动老五街片区旧城改造及城市修补、生态修复工作，全力推动290个海绵城市项目、15条地下综合管廊、29条市政道路、玉溪大河三期建设，完成红龙路改扩建、火车站站前广场及周边路网建设，新增城镇燃气管网53公里、推广居民用户1.5万户。加快县城和重点镇建设发展，打造澄江、峨山2个提质扩容示范县城，统筹推进海绵县城、海绵小镇、海绵乡村建设，搞好寒武纪小镇等18个特色小镇建设。推进产城融合，抓好高新区国家产城融合示范区试点，科学布局园区产业，以城聚产、以产兴城、产城联动、融合发展，带动人口集聚，促进农业转移人口市民化。出台加快建筑业发展实施意见，加强施工企业监管，确保建筑业增加值增长25%以上。

　　改善人居环境。增强管理意识，创新治理方式，改善城乡面貌。全面推进“六城同创”，确保国家节水型城市申报成功。严格执行《云南省玉溪城市管理条例》，提高精细化、规范化管理水平，着力解决交通拥堵、占道经营、私搭乱建等问题。全面实施提升城乡人居环境行动计划，推进城市“四治三改一拆一增”综合整治，加快中心城区生活垃圾焚烧发电、中水回用等项目建设，抓好4条黑臭水体和3条河道治理，改造棚户区1万户、完成8399户。推进农村“七改三清”环境整治，实施“百村示范”60个、“千村整治”605个，启动危房改造4900户、完成1.59万户，农村改厩5万户，抓好28个国家传统村落建设。新建改建城市公厕80座、农村公厕159座。加快增绿添色、点亮玉溪工程建设，实施“三湖”流域、红塔区全域绿化，安装太阳能路灯5万盏。推行统规联建，规范建房行为，改善居住环境，建设美丽乡村。

　　（八）强化生态环境保护

　　突出抓好湖泊保护治理。坚持“五个坚定不移”，严格执行“三湖”保护条例，加大工程治理和非工程措施力度，确保抚仙湖总体水质稳定保持Ⅰ类、星云湖和杞麓湖水质有所改善。完成“三湖”水污染“十三五”规划项目前期工作，确保开工率达50%以上。着力实施抚仙湖“四退三还”工程，加快推进沿湖2.8万人生态移民搬迁，下决心全部退出一级保护区内12户中央、省属企事业单位，启动湖滨区生态恢复工程，加快建设北岸生态湿地，搞好面山绿化，完成生态调蓄带二期等项目建设。加快星云湖环湖截污及底泥疏挖处置、杞麓湖环湖截污及农田减肥增效、通海第二污水处理厂及配套管网工程建设，完成两湖周边34个村落环境整治。积极推进抚仙湖、杞麓湖国家湿地公园试点建设。加快“三湖”径流区产业结构调整，发展环湖生态循环农业，减少农业面源污染。稳妥推进大小矣资搬迁工作，抓好东风水库、飞井水库等重要水源地保护。

　　全面推进生态建设。严格落实中央环保督察要求，确保问题整改到位、责任追究到位，严防问题反弹。加强森林防火，划定并严守林业生态保护红线，搞好陡坡地、石漠化综合治理，完成营造林18.6万亩、低效林改造4万亩。推进曲江、绿汁江、戛洒江流域水污染防治，治理水土流失面积281平方公里。开展土壤环境质量调查，开发整理土地2.3万亩，确保永久基本农田土壤环境质量不下降。深入推进大气污染防治，淘汰中心城区燃煤锅炉，加强施工现场环境监管和机动车尾气治理，基本淘汰黄标车，确保环境空气质量优良率达96%以上。持续推进生态文明县、生态文明乡镇和绿色创建工作。

　　切实加强节能减排。实施工业、建筑业等重点耗能领域能效提升计划，抓好污染减排工作，确保年度节能减排任务完成。加强重点污染源和污染隐患监管整治，稳步开展环境污染第三方治理。加强环境保护执法监管，抓好危险废弃物、重金属污染防治，确保重点项目建设环评率和“三同时”执行率均达100%。

　　（九）着力增进民生福祉

　　加强就业社保工作。实施创业促进就业工程，落实“就业创业玉溪”行动计划，新建3个创业孵化平台，新增城镇就业2.5万人，下岗失业人员再就业9000人。实施城乡居民增收工程，完善机关企事业单位工资增长机制，加大低收入群体和农民增收扶持力度，拓宽农村居民增收渠道。实施社保扩面提标工程，推进城乡居民医保整合，抓好重点领域工伤保险、老年人意外伤害保险扩面，以农民工、“农转城”人员、非公组织从业人员等为重点扩大社会保险覆盖面，城乡低保标准提高10%。搞好被征地农民养老保障。完善社会救助制度，搞好救灾救济。

　　统筹发展社会事业。实施教育提质惠民工程，完成二期学前教育三年行动计划和“全面改薄”建设任务，率先在全省实现义务教育基本均衡目标；进一步提高教育教学质量，逐步扩大高中办学规模，支持高等院校和民办学校发展，依托国内外知名院校和企业集团，积极开展创办互联网大学、足球学校等前期工作。实施健康养老养生工程，启动健康城市试点，发展健康养老业，整合中心城区医疗资源，规划建设北片区健康产业园，加快市儿童医院建设，推进市中医院易地搬迁和妇产医院建设前期工作，确保市医院改扩建、市急救中心投入运行，完成2个县中医院、2个乡镇卫生院、10个村卫生室建设，推动与中山大学合作创办三级医院；推进峨山医养结合试点县建设，完成5个乡镇敬老院改扩建、20个居家养老服务中心建设。实施人口均衡发展工程，落实“全面二孩”政策，关爱妇女儿童健康，健全扶残助残服务体系，广泛开展全民健身活动，办好各类体育赛事。健全公共文化服务体系，加快文化广播影视传媒中心建设，建成3座广播电视高山台；启动6个乡镇综合文化站建设；抓好文物文博和非物质文化遗产传承保护，启动中国最佳楹联文化城市创建工作。办好第五届聂耳音乐（合唱）周。抓好红十字、关心下一代、残疾人等事业，加强外事侨务、保密、档案、史志等工作。

　　促进社会和谐稳定。全面推进依法治市，全面开展“七五”法治宣传教育。加快民族团结进步示范区建设，全力维护民族团结宗教和顺。启动市综合检验检测院建设，加强食品药品安全监管。认真贯彻中央关于推进安全生产领域改革发展的意见，严格落实企业主体责任和政府监管责任，建立大检查大排查长效机制，继续深化打非治违，坚决防范重特大事故发生。加强防灾减灾和应急处置能力建设。加强基层矛盾调解组织建设，做好信访维稳工作。加强社会信用体系建设，完善社会治安防控体系，强化反恐维稳、缉枪治爆、禁毒防艾和反邪教工作，严厉打击非法集资、电信网络诈骗等违法犯罪行为，争创平安云南示范区，创建全国“长安杯”。推进国防教育、国防动员、民兵预备役和人民防空建设，创建云南省第十届双拥模范城。

继续办好十件惠民实事。

　　（十）加强政府自身建设

　　新形势新任务新常态，考验着政府的作风和能力。我们将牢固树立“四个意识”特别是核心意识、看齐意识，以更严的标准转变作风，更强的担当履职尽责，更实的举措优化服务，凝聚跨越发展正能量，提振干事创业精气神，增强政府执行力和公信力，努力建设人民满意政府。

　　严格依法行政。坚决服从市委领导，不折不扣执行好市委的各项决策部署。自觉接受市人大及其常委会的法律监督、工作监督和市政协的民主监督，广泛听取各民主党派、工商联、无党派人士和人民团体的意见建议，认真办理人大代表建议和政协提案。落实玉溪市法治政府建设实施方案，严守重大行政决策程序规定和终身责任追究制度，推进依法科学民主决策。完善学法用法制度，提高政府公职人员法治素养。强化权力运行监督制约，推行行政决策、管理、服务、结果“四公开”。做好地方立法和政府规章制定工作，加强行政执法监督，严格规范公正文明执法，建设法治政府。

　　加强能力建设。坚持以人民为中心，厘清政府与市场、社会边界，全面履行政府职能。加强公职人员学习培训和教育管理，弘扬玉溪精神，打造过硬班子、过硬队伍。强化公共资金、项目资金、国有资产等的监督管理，盘活存量、优化增量，最大程度发挥公共资源使用效益。规范政府购买服务行为。加快推进“互联网+政务服务”，优化服务流程，创新服务方式，提高服务质量，建设服务政府。

　　强化作风建设。严格遵守《关于新形势下党内政治生活的若干准则》《中国共产党党内监督条例》，始终把纪律和规矩挺在前面。健全正向激励机制和容错纠错机制，大力倡导敢于负责、勇于创新的担当精神，始终保持攻坚克难、奋力争先的进取精神，全面治理不敢为、不会为、不想为的庸懒散行为。强化工作落实，多出措施少发文，多下基层少开会，多抓落实少空谈，多解难题少抱怨，形成愿干事、能干事、干成事的良好氛围。强化督查督办，建立整改问责长效机制，落实敬业有功、怠业必惩各项规定，加强绩效管理，建设效能政府。

　　大力反腐倡廉。认真落实党风廉洁建设责任制，切实加强领导干部廉洁自律工作，营造愉快高效、风清气正的工作氛围。严格落实中央八项规定精神和省、市实施办法，严防“四风”反弹，坚持厉行节约，严控“三公”经费，一般性支出压缩20%。深化“六个严禁”专项整治，全面推进“阳光权力”工程，严肃查处工程建设、土地征用、矿产资源开发利用、国有资源处置等重点领域腐败行为。加强行政监察，推进审计全覆盖，坚决纠正侵害群众利益的不正之风，从严查处违法违纪案件，坚定不移惩治腐败，建设廉洁政府。

　　各位代表，新的一年，我们面临的任务艰巨而繁重，肩负的责任重大而光荣。让我们更加紧密地团结在以习近平同志为核心的党中央周围，在中共玉溪市委的坚强领导下，迎难而上，砥砺前行，扎实抓好经济社会发展各项工作，以优异成绩迎接党的十九大胜利召开！
